# Case of Indurating Inflammation of Liver and Acute Desquamative Nephritis

**Published:** 1878-04

**Authors:** W. T. Belfield


### CASE OF INDURATING INFLAMMATION OF LIVER AND ACUTE DESQUAMATIVE NEPHRITIS.

By Dr. Belfield.

H. H——, 72 years old, was admitted to the hospital February 5, 1878. He had enjoyed excellent health, free from any taint, inherited or acquired, until three months previously, when he fell a victim to ague. For five weeks he shook daily, and then obtained relief.

Three weeks later he one day suffered a sharp pain in the right hypochondrium, and became very feverish. In a few days the severity of the pain and fever had somewhat subsided, but he had never regained sufficient strength to leave his bed.

Four days prior to admission he felt a sharp pain again over the liver, and soon his abdomen began to enlarge.

On admission he was well-nourished, his functions well performed; his bowels moved daily, the stools being soft and brown. The abdomen was much distended, partly by liquid and partly by gas; the liver dulness was imperceptible below the seventh rib. There was marked tenderness in the right and left hypochondriac regions. There was some œdema of the lower lobe of the right lung, and slight swelling of the feet. The heart sounds were normal. Pulse 120, temperature 103½.

The urine was of light brown color and low specific gravity, containing about ten per cent. of albumen, numerous small epithelial and granular casts, and much loose renal epithelium.

Feb. 7.—Intense jaundice has been developed.

Feb. 9, 10 and 11, he suffered from constant hiccough, the other symptoms remaining unchanged, except that his pulse and temperature were about normal.

Feb. 13.—He had a chill, followed by headache, fever and vomiting.

Feb. 14.—Another chill, with severe pain across the loins.

Chills occurred daily on the 15th, 16th and 17th. The general symptoms did not change much, though he was evidently losing strength.

Death occurred Feb. 21. An examination showed considerable transudation into the peritoneal sac as well as into the general connective tissue of the lower extremities.

The spleen was some ten inches long, five inches broad, three and a half inches thick; its consistence was unusually firm.

The kidneys were enlarged, and in their medullary portions at least, hyperæmic. The liver was slightly but uniformly decreased in size, and deeply stained with bile; its surface presented numerous elevations, varying in size from a pin's head to a bean. The tissues generally were deeply tinged with biliary coloring matters.

Microscopic examination of the kidney revealed an excellent example of "cloudy swelling." The granular, swollen, oblong epithelial cells nearly obliterated the calibre of the dilated tubules. The nuclei were generally somewhat indistinct.

The microscopic anatomy of the liver, however, was especially interesting. Here were the evidences of that indurating inflammation which follows long-continued or oft-repeated hyperæmia of the organ. The liver-cells were, on the average, perhaps a little larger than normal, but were highly granular, the granules having in some instances coalesced so as to form fat drops, which lay in and around the cells.

The interlobular spaces were extremely broad, their breadth having been attained, apparently, at the expense of the lobuli, which were correspondingly diminished in diameter.

The interlobular spaces consisted of spindle-cells, fibers and round, indifferent cells. The spindle-cells were usually observed in the central portions, near the portal vessels. Mingled with these, and extending beyond them toward the lobules, were thick straight fibers. Scattered over the entire interlobular space, but collected in great numbers at the margins of the lobules were the small round cells.

These two cases exemplify the first two stages of indurating inflammation of the liver. First as to the liver-cells: The former case showed what Virchow calls a "nutritive irritation,"—an incitation of the cells to appropriate an abnormal amount of nutriment, resulting in the coagulation of certain albuminous constituents of the protoplasm and their appearance as granules. In the latter case reported, these granules had met the inevitable fate of imperfectly-nourished protoplasm and had undergone fatty degeneration, involving, in some instances, the entire cell in their ruin. Second as to the interlobular spaces: In the former case reported, these were filled almost exclusively with small, round, indifferent cells, the products of formative irritation of nuclei as well as of migration from vessels. The few fibers scattered through the mass, however, indicated conclusively that the tendency of these young cells was toward development; that the continuance of the process would have resulted in the formation of connective tissue.

In the second case reported, time had been allowed for the continuance of the process, and there had resulted the formation of the closely-packed spindle-cells, and the long straight fibres which surrounded the vessels. That the developing process had not ceased was proved by the presence, at the margins of the lobules, of myriad small cells capable of a similar development and further encroachment upon the acini. It is interesting to note that the microscopic appearances fulfill the expectations which might be based upon the clinical histories of the respective cases. In that of the woman, the morbid process had not, probably, existed more than ten days, nor was there any history of a previous hyperæmia exceeding physiological limits; hence the morbid appearances were those of a very early stage in the departure from normal action. In the second case, however, there had been daily an active hyperæmia of the liver (perhaps beyond the physiological limit) during the five weeks of his suffering from ague; and there had been, three weeks later, a decidedly pathological engorgement of the organ. Hence it is highly probable that the attack which immediately preceded his admission to the hospital was a mere exacerbation of a process which had begun several weeks previously. The morbid appearances, therefore, were those of an advanced and advancing stage.

